# A study on the applicability of scoria gravel an alternative base course material through blending with marble waste aggregate

**DOI:** 10.1016/j.heliyon.2022.e11742

**Published:** 2022-11-21

**Authors:** Multazem Mohammed, Anteneh Geremew, Murad Mohammed, Abubekir Jemal

**Affiliations:** a Madda Walabu University, Department of Civil Engineering, Bale-Robe, Ethiopia; b Jimma University, Faculty of Civil and Environmental Engineering, Jimma, Ethiopia

**Keywords:** Scoria gravel, Marble waste aggregate, Crushed stone aggregate, Percentage by weight

## Abstract

This study aims to evaluate the applicability of Scoria gravel as an alternative base course material in flexible pavements through blending with marble waste aggregate (MWA) by modifying the physical and mechanical engineering properties of scoria. Non-Probabilistic sampling techniques and experimental methods were used. To achieve the objectives of the study, the laboratory tests were passed in three steps. First, the Engineering properties of materials were independently tested; the result proves the marginality of scoria gravel. Second, scoria gravel was mechanically stabilized by 20% percentages by weight increments of MWA. The obtained engineering properties test results at 20:80 Scoria to MWA mix ratios are 2.56%, 21.38%, 18.59%, 19.27%, 17.45%, 13.77%, Non-Plastic, 1.21%, and 73.4%, for Specific Gravity, Aggregate Crushing Value, Aggregate Impact Value, Loss Angeles Abrasion, Flakiness Index, Elongation Index, Atterberg's limit, Water Absorption, and California Bearing Ratio (CBR) respectively. These test results fulfilled the ERA standard specification for GB2 and GB3 base course materials. However, the CBR test results showed a failure to meet the standard spécification. Thus, 20:80 Scoria to MWA percentage by weight ratio was selected as a control mixture. So, Crushed Stone Aggregate was added at 5% percentage by weight to improve the CBR of scoria gravel. Therefore, the CBR value of 82.13% attained the ERA standard Specification limit for base course materials at 15:60:25 percentages by weight ratio of scoria gravel, MWA, and CSA respectively. Finally, Based on this study it was recommended to use scoria gravel as an alternative base course construction material, when it was found abundantly near construction vicinity.

## 1 Introduction

Through connections with airports, railway stations, and ports, roads play an essential part in the development of intermodal transportation. Road construction is a crucial concern in civil engineering practice for any country's to initiate a rapid industrial development, increases socio-economic intractions [1, 2, 3]. This is because transportation infrastructures allow people and supplies to move quickly and easily from one location to another at required time, enhancing social mutual interaction. A large-scale construction of transportation infrastructure projects have historically had major negative environmental consequences around the world [4]. The majority of current road construction procedures rely heavily on naturally occurring aggregates minerals sourced from mines. This mineral aggregates that are the most mined in construction industrial materials in the world with extractions sites located near every project site. Globally, it was estimated that 32–50 billion metric tons of aggregates (sand and gravel) are extracted each year for various purpose [5]. The extraction of these aggregates from their natural sources depletes its natural resources and pollutes the environment on a large scale that is leading to environmental degradation [6, 7, 8]. In flexible pavements the role of the unbound layer is unique, its primary function is to enhancement of structural layer capacity. To strengthen structural capacity, the base course must be able to resist deformation owing to loading [9]. In road infrastructure, the quality of the base course material in terms of a significant engineering properties is serious. If the required base course material is not available within an unacceptable distance of the construction site, then high rates must be paid during the road construction process, causing substantial delays [8]. In such instances, working with low-quantity materials that are readily available locally is a good option. Scoria is a volcanic product that is abundantly available in various parts of the world such as; Turkey, Algeria, Papua New Guinea, Syria, Saudi Arabia, Cameroon, Indonesia, Rwanda, Tanzania, Yemen, Ethiopia, and many other subtropical countries [10]. In Ethiopia, Scoria gravel is extensively found especially in the Great Rift Valley, which crosses the northeastern part of the country including Akaki, Bishoftu, Mojo, Lake Ziway, Lake Chamo, Woliso, Butajira, Bekoji, Loggia, and Adama [11, 12]. Because of its lightweight, rough circular surface, and high porosity, scoria gravel is vulnerable to compaction. Additionally, the lack of finer materials in comparison to standard specifications, as well as weak, easily broken particles, make scoria gravels unsuitable for the usage of base course construction [13]. Moreover, Marble is one of the largest manufactured natural stones in the world and it accounts for 50 percent of the world's natural stone production. Depending on the type, quarrying, and processing method used and its irregular shape, smaller size, cutting and polishing process, marble production generates 20–30% waste [8]. Ethiopia has presently about fourteen marble processing Industries located in various parts of the country, which generated 409,374.00, 572,421.00, 613,820.00, 770,000.00, and 1,000,000.00 m^3^ of marble commodities in 2009, 2010, 2011, 2012, and 2013, respectively [14]. Surprisingly, this waste is dumped on open land, which creates a lot of environmental as well as health problems. The following environmental damage might occur: porosity and permeability of topsoil are reduced enormously, the fine marble particles with high pH reduce the fertility of the soil, causing air pollution, corroding nearby machinery, depleting natural resources in terms of wastewater, small pieces of marble, erosion of top fertile soil cover, contamination of the rivers and other water, vibrations and noise effects which, associated with quarry operations of drilling and blasting [15]. This research was carried out using scoria gravel taken from a quarry site founds in Adama Area, Oromia Regional State. The study aims to encourage the usability of locally available marginal low-quality materials and industrial waste materials like Scoria Gravel and Marble Waste Aggregate to address the concerns linked to the scarcity of standard materials and environmental degradation in the study area. As a result, the construction industry in the study area has benefited from using abundantly available resources rather than expensive virgin materials, implying that natural resources will be conserved. The laboratory investigations were conducted at Jimma Institute of Technology (JIT) highway engineering laboratory. These include:- Sieve Analysis, Aggregate Crushing Value, Ten percent Fine Value, Aggregate Impact Value, Loss Angeles Abrasion Value, California Bearing Ratio, Compaction, FI, EI, Specific Gravity, and Water Absorption tests that were used to investigate the materials in the laboratory.

### 1.1 Statements of the problem

In flexible and rigid pavement, natural aggregate is one of the most important components matrials utilized in constructing various layer morethan 95% of the total ingradients. The increasing consumption of aggregate by the construction industry has led to the rapid depletion of natural aggregate which is non-renewable energy and a good quality standard material is readily grows scarcer [6, 16]. There are potential environmental impacts associated with aggregate extraction including the conversion of land use, changes to the landscape, loss of habitat, noise, dust, blasting effects, erosion, and sedimentation [6]. Most of the environmental impacts associated with aggregate mining are relatively benign. However, extracting aggregate from some areas may alter the geologic conditions, which, in turn, the dynamic equilibrium of the area, resulting in cascading environmental impacts [6, 17]. On the other hand, a significant amount of waste is generated with increases in the demand for landfill sites, which negatively affects the environment [18]. Hence, instead of producing aggregates and minerals from virgin sources, it is more than appropriate to use waste products from industries and locally abundant low-quality materials. Previous researchers conducted a variety of studies on marble waste and scoria as substitutions of different road construction materials i.e. subgrade, sub-base, base course, and as fine aggregate for asphalt filler individually or through blending with other conventional or unconventional materials. But no research has been done on combining both marble waste and scoria as alternative base course materials. So, through blending the two non-conventional materials the reliance on sustainable development, Ethiopia's green legacy agenda of GTP III of green Industrialization, as well as maintaining the Green House Gas (GHG) emission issue of the world, due to extraction of conventional aggregate for infrastructural development can be reduced. Therefore, the main objective of this study was to investigate the applicability of scoria for base coarse material through blending with waste marble aggregate.

## 2 Materials and methods

### 2.1 Material required

All materials used in the investigation were extracted from quarries near Adama, which is 100 km far from the capital city of Ethiopia, Addis Ababa along the road connects the **eastern parts of the country towns and Djibouti ports.** Non-probable purposive sampling techniques were adopted to collect materials used for conducting this research method.

**Figure 1:**
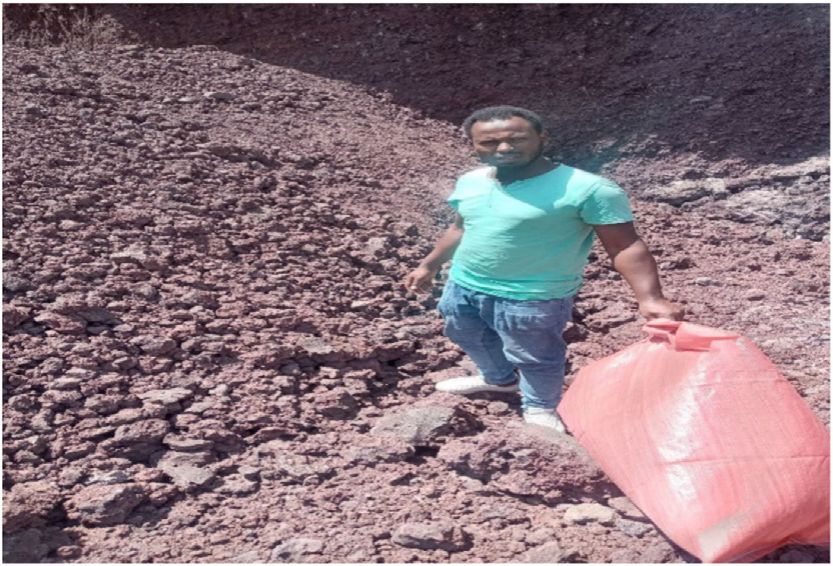
Scoria quarry site near Adama town.

Scoria is a volcanic product, which generally has a rough surface and highly porous nature. The samples of scoria gravel have been taken from the quarry site found at 5 km far from adama town along the main road of Arsi Asalla. The color of scoria gravel were varies from light brownish to dark brownish as shown in Figure 1.

**Figure 2:**
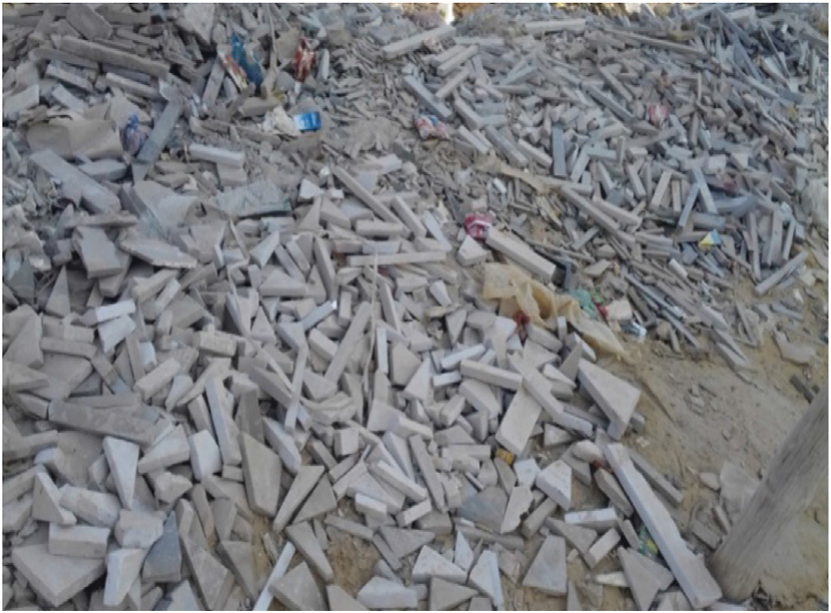
Marble waste.

Marble is defined as a recrystallized medium-to coarse-grained carbonate rock composed of calcite or dolomite, or calcite and dolomite. The industrial marble waste was collected from Cesso marble processing industry as shown in Figure 2, found at Adama city which acquired a raw marble from Shenila quarry 550 km far from Addis Ababa.

**Figure 3:**
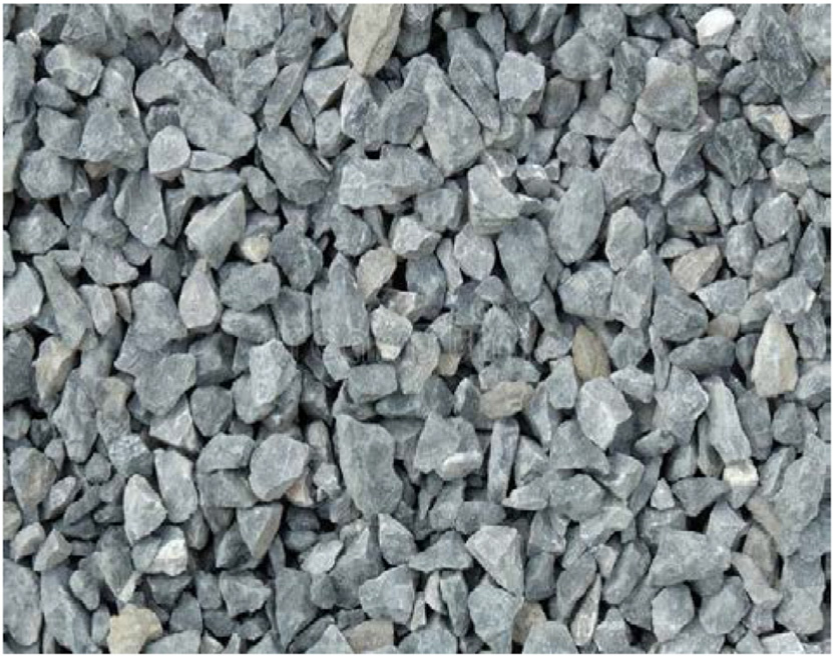
Crushed stone aggregate.

Crushed stone aggregate (CSA) is natural crushed rock materials, gravels, and sands or slag aggregates are the basic raw material used by construction industries as shown in Figure 3.

### 2.2 Methods

The research was conducted at Jimma University, Jimma Institute of Technology (JIT) in the Highway engineering laboratory. The materials used in the investigation were described concerning their source and physical properties. The physical and mechanical engineering properties of aggregate were held constant in all the test procédure described in Table 1, while the amount of scoria gravel, marble waste aggregate, and Crushed stone aggregate were variable.

**Table 1:** Tests on aggregate and test methods.

Type of Aggregate Test	Adopted Test Methods/Designation
Particle size distribution/sieve/	AASHTO T-27-93
Specific Gravity (Apparent),%	AASHTO T-85, AASHTO T-84, & ASTM C-127, C-128
Water Absorption,%	AASHTO T-85
Flakiness Index,%	BS812 Part 105-1990
Elongation Index,%	BS812 Part 105-1990
Aggregate Crushing Values,%	BS 812 Part-110
Ten Percent Fine Values, KN	BS 812 Part-111
Aggregate Impact Values,%	IS 2386-Part-4
Los Angeles Abrasion Test,%	AASHTO T96
Compaction	ASSHTO-D1557
California Bearing Ratio,%	AASHTO,T 193-93

### 2.3 Equipment

There are various equipment was used in labouratory works as per the standerd specification to achive this study as illustrated in Table 2.

**Table 2:** Lists of equipment and functions.

S/N	Equipment	The function of the equipment's
1	Sample splitter	➣ Used to reduce field sample of aggregate
2	Abrasion Los Angeles machine https://www.google.com/aclk?sa=l&amp;ai=DChcSEwiHyJ_n2-X3AhXoDIsKHYQhCSYYABAAGgJlZg&amp;ae=2&amp;sig=AOD64_29E7Qj_yjQNNhAO6aKsShPZQphoQ&amp;q&amp;adurl&amp;ved=2ahUKEwiFhZfn2-X3AhWN4YUKHdJxAgwQ0Qx6BAgCEAE	➣ Abrasion is a measure of the aggregate resistance to wear or hardness.
3	Compression testing machine	➣ Used to measure the resistance of aggregate crushing under gradually applied compressive load.
4	Impact testing machine	➣ Measures of the resistance to sudden shock or impact load
5	Different size sieve	➣ Used to measure the particle size distribution of the aggregate sample.
6	Loading machine of 50 kN capacity fitted with a calibrated proving ring to which plunger has to be attached.	➣ Used to measure the penetration capabilities of materials.
7	Shape test gauge	➣ Used to measure the flakiness and elongation properties of aggregate
8	Weighting sensitive balance, sample container, pycnometer, and water bath	➣ Used to measure the specific gravity of sample specimen

### 2.4 Study design

**Figure 4:**
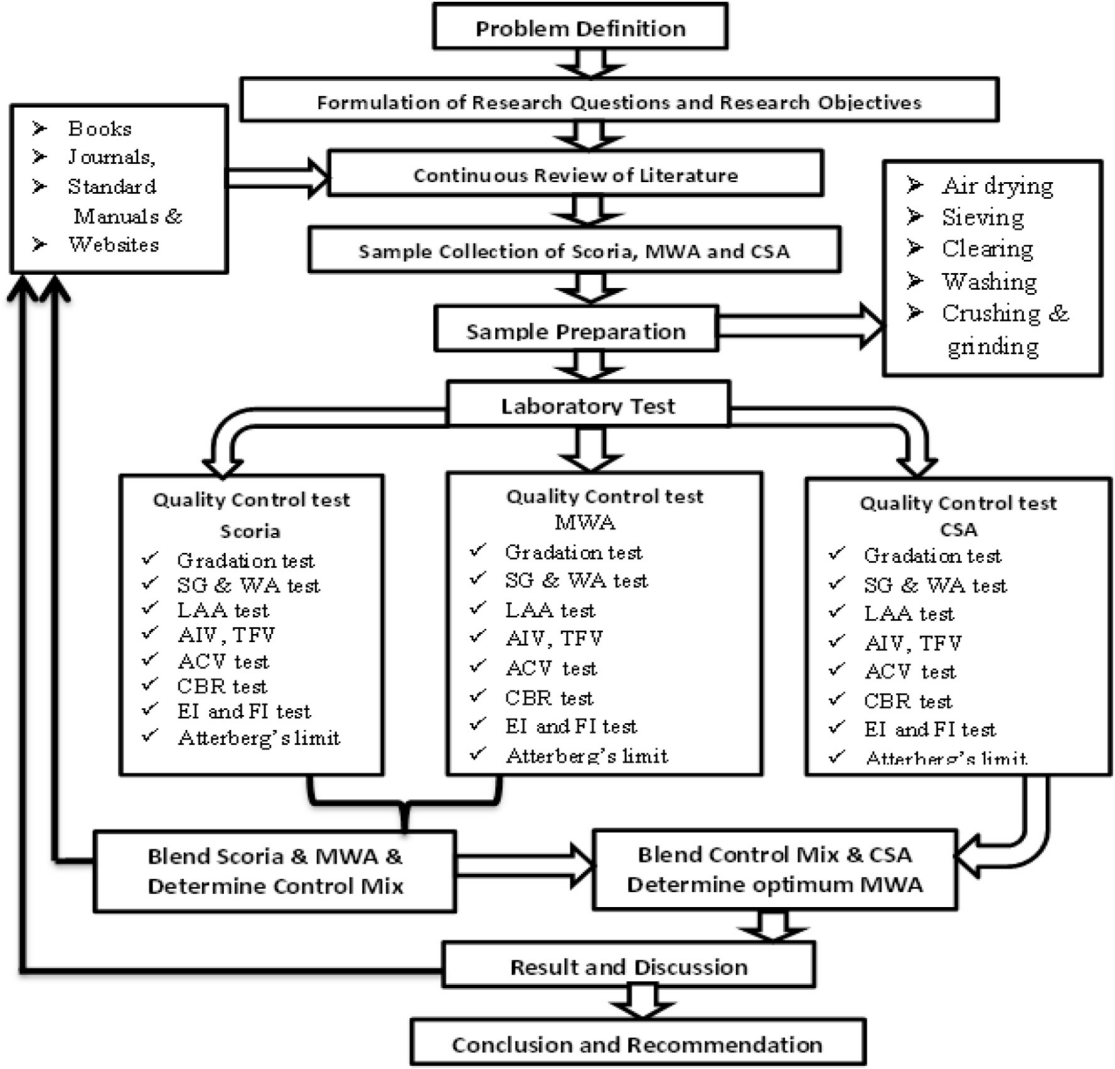
Flow chart showing study design.

An experimental comparative study design was employed in the current study. An experimental comparative study design was employed in the current study. Figure 4 shows a Flow chart for the research design to conduct laboratory tests such as the physical and mechanical properties of Scoria Gravel, MWA, and CSA materials, determining the effect of Scoria gavel on quality requirements of base course material and blending MWA with CSA to find out possible replacement amount that satisfies the requirement of the ERA manual standard specification and by gradation requirement for base course material.

### 2.5 Sampling techniques and procedures

**Figure 5:**
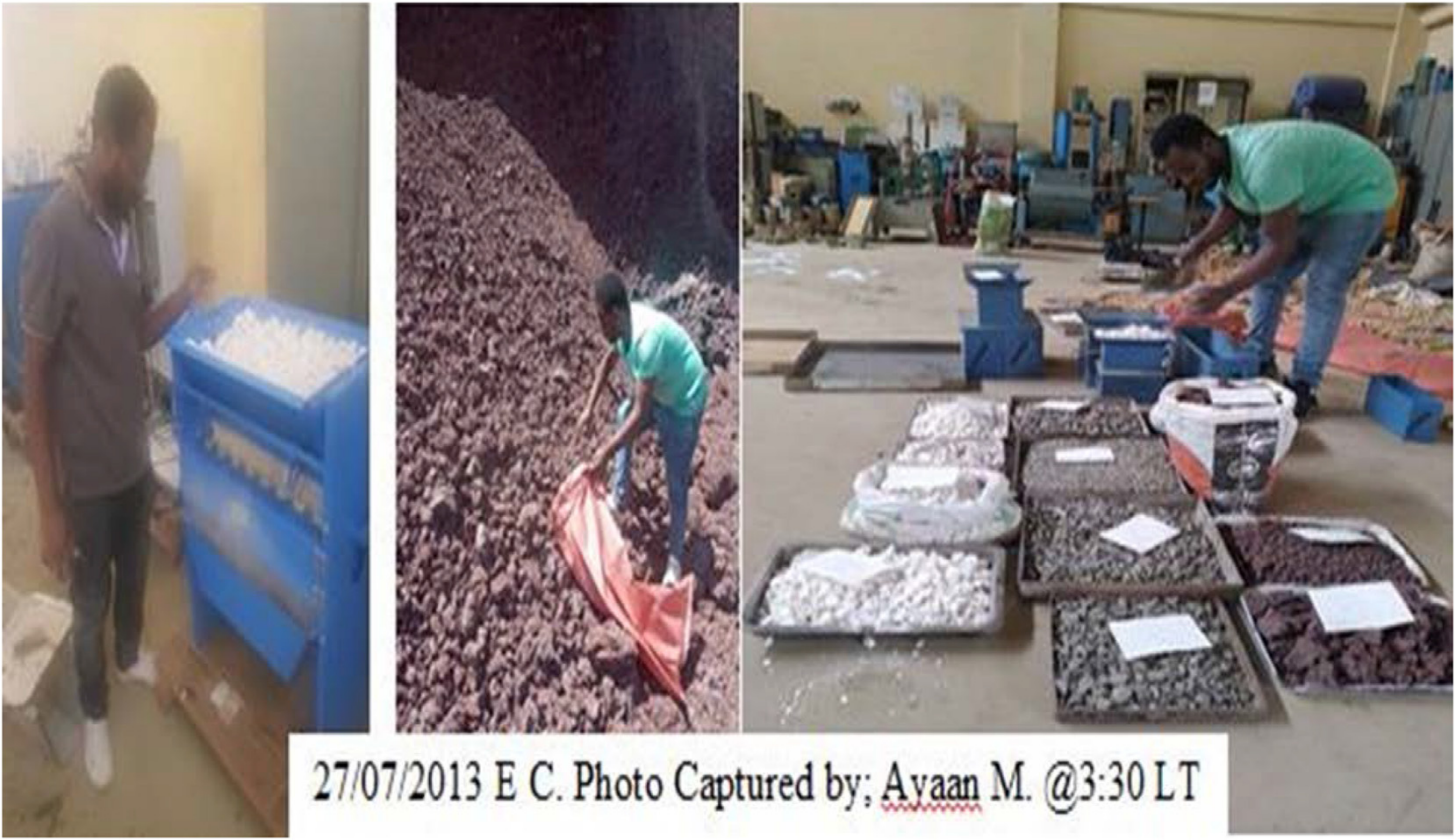
Shown while sample collection and preparation were performed.

The Sampling techniques adopted for scoria gravel, marble waste aggregate, and crushed stone aggregate were Purposive sampling techniques which is a non-probability method that involves the selection of samples for laboratory analysis. The samples were collected according to the procedure AASHTO T-2 Methodology for sampling from stockpiles and reducing samples of aggregate to testing size was according to AASHTO-T248. Figure 5 shows the sampling activities for each test, quartering, riffle splitter, and weighting are used for sampling techniques.

## 3 Results and discussion

### 3.1 Properties of scoria gravel, MWA, and CSA

**Figure 6:**
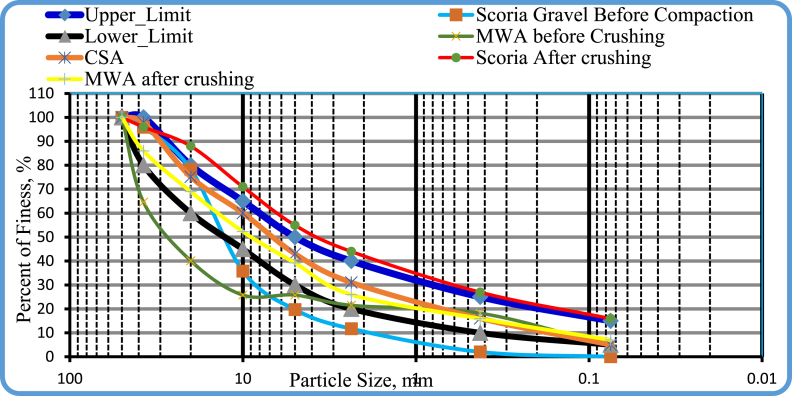
Scoria gravel, MWA, and CSA before and after crushing particle size distribution curve.

In Figure 6 shows; Particle size distribution curves before and after preparations. Thus, the line plotted for scoria gravel before crushing illustrates the materials contain high amounts of coarse-grained particles, and the line representing scoria after crushing reveals that the materials were vulnerable to compaction. Since the line stands for scoria gravel before crushing was lies below the lower boundary and scoria gravel after compaction lies above the upper boundary limit. In principle, if the plotted particle size distribution curves of any material pass in between the upper and the lower limit boundary line, it can be considered as the material that has the best particle size distribution. Whereas, if the plotted line approaches the upper limit, most of the particles in the sample pass the maximum specified percentage of passing, and if the line goes tightly approaches the lower limit the sample contains more coarse-grained particles. The preparations of scoria gravel and marble waste aggregate through crushing increased the workability of materials for conducting the experimental investigations. From uniformity coefficient Cu and Curvature coefficient Cc, which were determined from the plotted line particle size diameter corresponding to the percentage of passes, 10%, 30%, and 60%, the gradation measures were determined, which were designated as D10, D30, and D60. The analysis result for gradation measures of scoria gravel, marble waste aggregate, and crushed stone aggregate were reported under soil classification.

**Figure 7:**
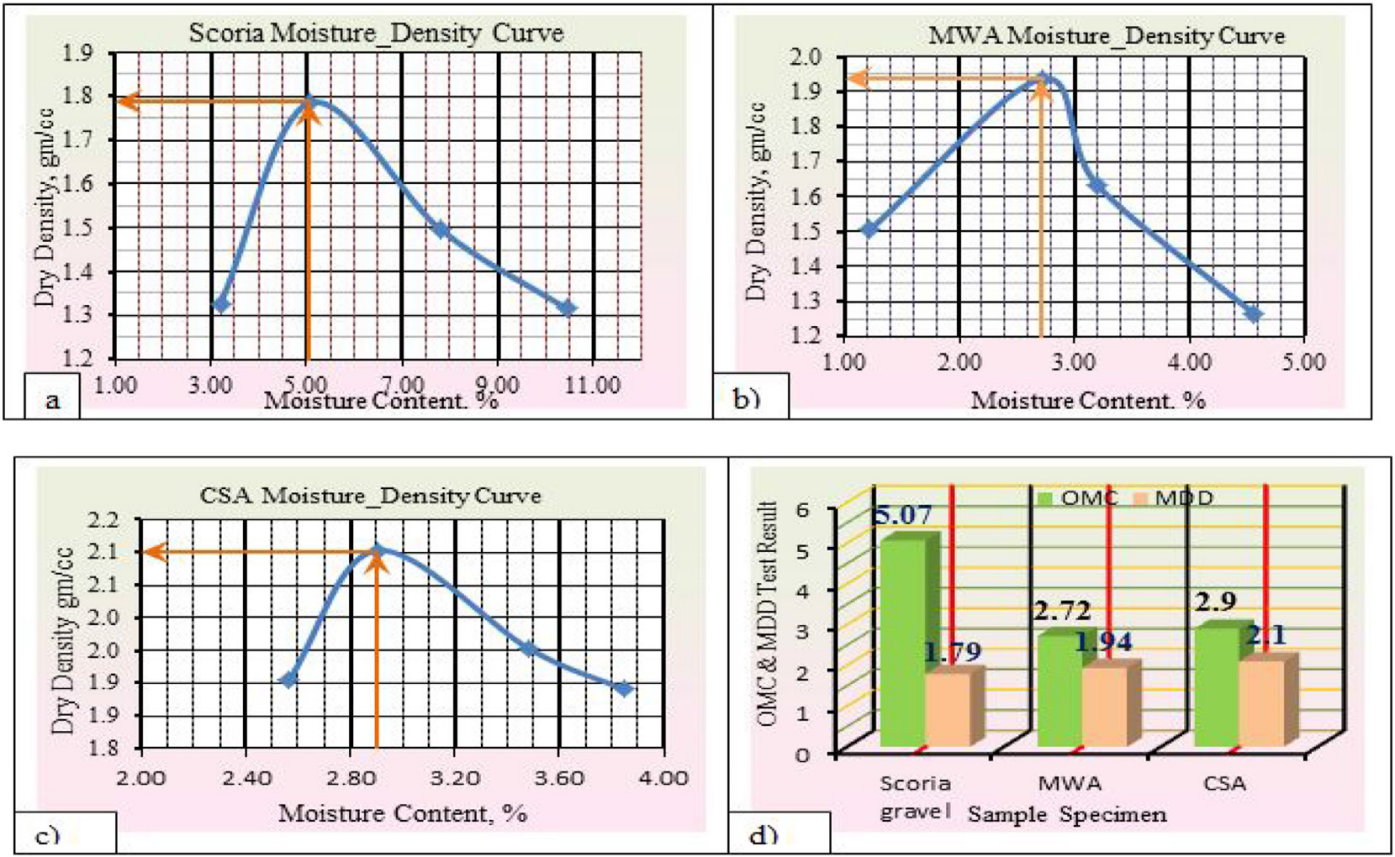
a) Scoria moisture density curve, b) MWA moisture density curve, c) CSA moisture density curve, and d) OMC and MMD relation diagram.

The detail laboratory investigation results are shown in Table 3 for water absorption, average bulk oven-dry specific gravity, average bulk saturated surface dry specific gravity and apparent average specific gravity of both coarse and fine scoria aggregates were 5.50%, 1.97, 2.08, 2.21, and 2.99%, 2.28, 2.21, and 2.37 respectively. The flakiness and elongation index obtained from laboratory tests for scoria gravel is 13.55% and 11.70%. While Flakiness and elongation index of MWA and CSA are 18.59%, 14.37%, 14.66%, and 13.63% respectively. Thus, both MWA and CSA samples were within the ERA standard specification for Base course construction materials. Obviously, the test result indicated in Table 3 shows that the aggregate crushing value was 34.01%, 19.70%, and 10.79% for Scoria gravel, Marble Waste Aggregate, and Crushed Stone Aggregate respectively. Therefore, scoria gravel and marble waste aggregate do not satisfy the minimum requirement for base course materials in dry conditions. The AIV test results show the test specimen fulfills the minimum requirements of ERA 2013, Standard Specification limit. The Loss angles abrasion values for scoria gravel, MWA and CSA were 26.6%, 17.44%, and 9.81% respectively. From compaction test result the OMC were 5.07%, 1.80%, and 2.90% while the MDD 1.79 gm/cm^3^, 1.94 gm/cm^3^, and 2.10 gm/cm^3^ for Scoria, MWA, and CSA respectively. The obtained soaked CBR for scoria gravel was 49.30% at 98% of compaction MDD which does not satisfy the ERA minimum requirements. But, MWA and CSA were given 85.7%, and 116.9% respectively, to satisfy the ERA standard specification requirements. The typical Moisture–Density Relation of Scoria Gravel, MWA and CSA as illustrated in Figure 7(a, b, c, d).

**Table 3:** Engineering property test result for neat scoria gravel, MWA, and CSA.

Engineering Properties		Aggregate Materials			ERA, BS, AASHTO, ASTM, IS Standard Limits
		Scoria gravel	MWA	CSA	
Specific Gravity (Apparent),%	Fine	2.37	2.7	2.78	2.5–3%, AASHTO T-84,85
	Coarse	2.21	2.64	2.74	
Water Absorption,%	Fine	2.99	0.17	0.17	<2%, AASHTO T-85
	Coarse	5.5	0.13	0.4	
Flakiness Index,%		13.55	18.59	14.66	<30%, BS 812 Part 105
Elongation Index,%		11.7	14.37	13.63	10–35%, BS 812 Part 105
Aggregate Crushing Values,%		34.01	19.7	10.79	<29%, BS 812 Part 110
Ten Percent Fine Values, KN	Dry	86.5	93.4	167.1	>110 KN, BS part 111
	Wet	70.2	87.4	143.3	-
	Dry/Wet	81	94	86	>75 KN, BS Part 111
Aggregate Impact Values,%		27.04	17.35	8.88	<25%, IS 2386-Part 4
Los Angeles Abrasion Test,%		26.6	17.44	9.81	<45%, AASHTO T-96 & ERA 2013
Compaction	OMC,%	5.07	1.8	2.9	NS
	MDD, g/cm^3^	1.79	1.94	2.1	NS
California Bearing Ratio,%	% CBR	49.3	85.7	116.9	>80%, AASHTO T 193-93 and ERA 2013
	Swell	0.03	0.01	0.01	

NS; not specified.

### 3.2 Classification of soil

From the test results of Scoria gravel, MWA, and CSA summarized in Table 4, all materials were classified as A-1-a since having less than 15% of fine contents and 16% for scoria after compaction, of particles passing a sieve opening size of 0.075 mm and zero PI. According to USCS, Scoria and marble waste aggregate before crushing, and crushed stone aggregate are classified as well-graded gravel with sand (GW) Since, the value of CU is greater than 4 and ∗∗∗. Whereas the marble waste aggregate before crushing has Cc = 22.85 and Cc = 159.60 which was described as poor graded materials with sand.

**Table 4:** Aggregate classification by using AASHTO and USCS.

Parameter used for classification	Aggregate Materials				
	Scoria gravel		MWA		CSA
	Before Compaction	After Compaction	Before Compaction	After Compaction	
D_10_ (mm)	2.021	0.051	0.21	0.192	2.34
D_30_ (mm)	8.200	0.766	12.95	3.169	2.228
D_60_ (mm)	15.693	6.569	34.23	14.633	9.987
Coefficient of Uniformity (Cu)	7.76	129.785	159.60	76.05	42.720
Coefficient of Curvature (Cc)	2.12	1.766	22.85	2.574	2.124
Gravel Content,%	80.29%	45.03%	74.22%	60.82%	56.97%
Sand Content,%	19.72%	38.97%	21.20%	32.18%	38.02%
Fine Content,%	0%	16%	4.57%	7.01%	5.00%
AASHTO Classification	A-1-a	A-1-a	A-1-a	A-1-a	A-1-a
USCS Classification	GP	SW	GP	GW- GM	GW

### 3.3 Engineering property determination of blending scoria gravel and MWA

#### 3.3.1 Particle size distribution

**Figure 8:**
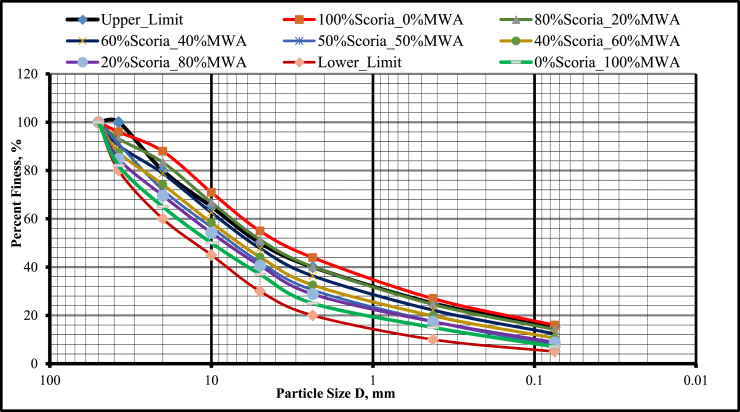
Blended scoria gravel and MWA particle size distribution curve.

As was observed in Figure 8, the mix proportion of 20% Scoria gravel-80% Marble Waste aggregate has a particle size distribution curve within the acceptable value of ERA for GB_2_ and GB_3_ as a base course material. Fineness modulus of course aggregate varies from 5.5 to 8.0. And for all aggregates or combined aggregates fineness modulus varies from 3.5 to 6.5 [9]. The fineness modulus of fine aggregate varies from 2.0 to 3.5 mm. According to this limitation, the aggregate mixes used in this research were not full coarse or fine, but the combination of the coarse and fine aggregate because fineness modulus varies from 3.63 to 4.19 which lies between 3.5 and 6.5 as presented in Table 5.

**Table 5:** Grading and fineness modulus of aggregate mixes used in this study.

Type of mixture	Grading Modulus, (GM)	Fineness modulus, (FM)
100% MWA	2.53	4.19
20% MWA_80% Scoria Gravel	2.40	3.63
40% MWA_60% Scoria Gravel	2.43	3.77
50% MWA_50% Scoria Gravel	2.45	3.83
60% MWA_40% Scoria Gravel	2.47	3.91
80% MWA_20% Scoria Gravel	2.52	4.11
100% Scoria Gravel	2.46	3.77

#### 3.3.2 Specific gravity and water absorption blended scoria gravel and MWA

**Figure 9:**
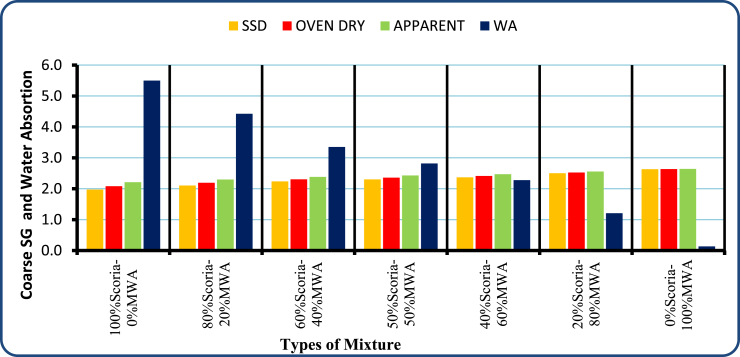
Test results of SG and WA for coarse aggregate test analysis.

**Figure 10:**
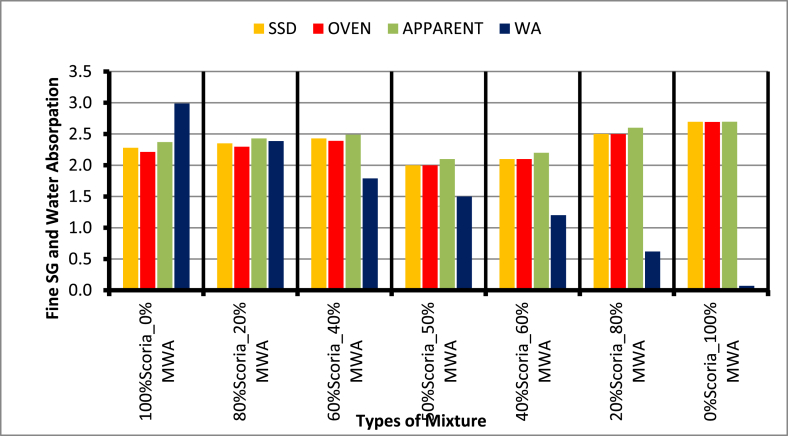
Fine aggregate specific gravity and water absorption test results.

From Figures 9 and 10 the specific gravity for neat Scoria Gravel has 2.21 and 2.63 for MWA. Thus, all the blended scoria gravel mixtures have a specific gravity between these values. Accordingly, most of the values obtained from the blended scoria gravel test result were less than the minimum ERA recommended values. But, the 20/80 blending proportion of scoria gravel to MWA satisfies the minimum recommended values with 2.56 for the course and 2.6 for fine material.

#### 3.3.3 Flakiness and elongation index for blended scoria gravel and MWA

**Figure 11:**
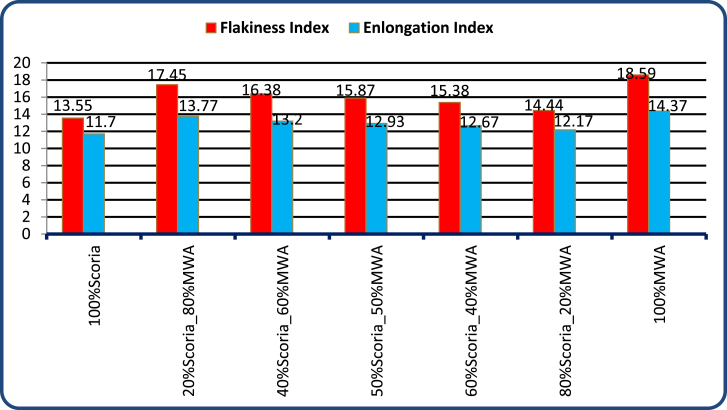
FI and EI test result of scoria gravel and MWA

As indicated in Figure 11 the percent of Scoria gravel increases the flakiness index also increases, but all mixes have value within the specification limits of ERA and BS standard specification that recommends maximum FI<30% and EI is between 10%–35% [9] and samples were prepared based on AASHTO T 2–91 method. Therefore, the blended aggregate has a flakiness index of 14.44%–17.45% and an elongation index of 12.17%–13.77% which was all the blended founds within the required standard specification limit.

#### 3.3.4 Aggregate crushing and ten percent fines value blended samples of scoria & MWA

**Figure 12:**
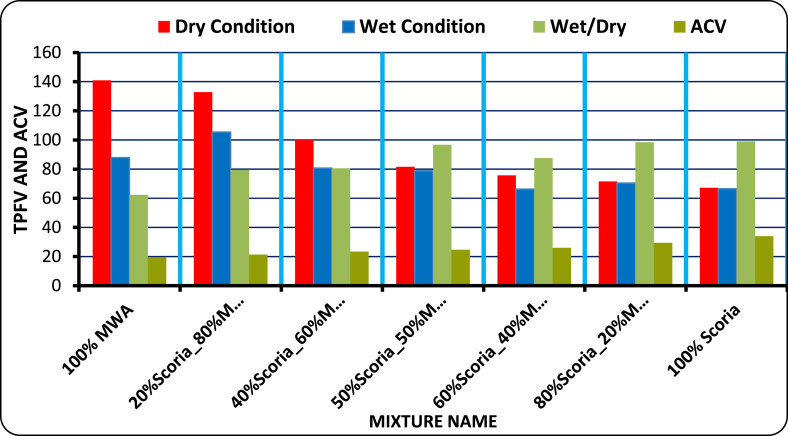
Tpfv and ACV of scoria gravel and MWA

Based on trest result shown in Figure 12, the required dry ten percent fine value was satisfied at 20/80 scoria to marble waste aggregate blending proportions 132.8 KN > 110 KN. Thus, the blending ratio satisfies at 20% Scoria–80% MWA mixing point, the rest blending proportions does not fulfill the requirement for dry condition, but the wet/dry ratio at all blending points satisfies the requirement of ERA standard Specification limit. This indicates that Scoria gravel has low strength material when compared to MWA concerning the resistance of the gradually applied load.

#### 3.3.5 Aggregate impact value (AIV) for blended scoria gravel and MWA

**Figure 13:**
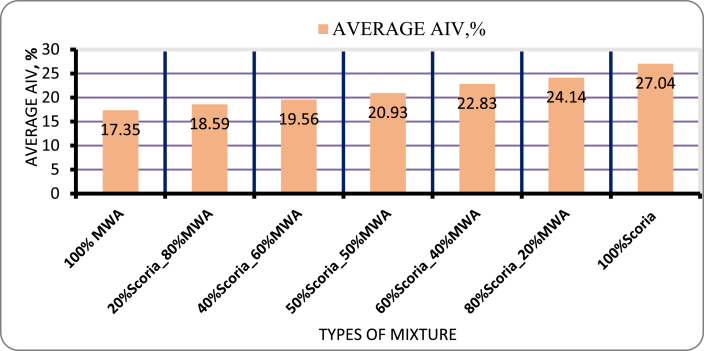
Aggregate impact value result for scoria gravel and MWA

As per BS 812: PART 112: 1990 the aggregate impact value is computed to find out the capacity of aggregate materials to resist sudden wheel applied load. The result of this study shown in Figure 13 indicates that the effects of the minimum impact value significantly increased with the addition of marble waste aggregate. In relation to this, aggregate impact value for blended scoria gravel ranges from 18.59%, 19.56%, 20.93%, 22.83%, and 24.44% aggregate after mix with, 80% MWA, 60% MWA, 50% MWA, 40% MWA and 20% MWA respectively.

#### 3.3.6 Los angles abrasion value (LAAV) for blended scoria gravel and MWA

**Figure 14:**
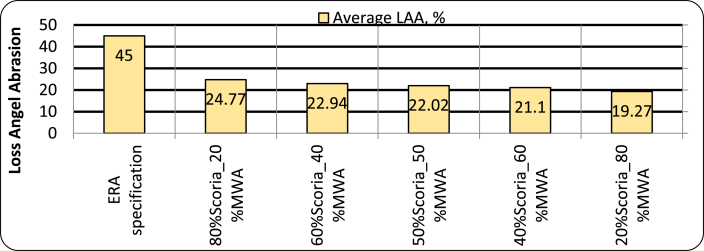
Loss Angeles Abrasion Test Result for blended Scoria Gravel and MWA.

ERA 2013 Specification states that the maximum Abrasion Value for aggregate used for base course material is limited to 45% [9]. As shown in Figure 14 the mixture containing the maximum amount of marble waste aggregate has a lower abrasion value than the mixture with the maximum amount of Scoria gravel. As the Los Angeles abrasion value goes decreases, the obtained values can be interpreted as maximum resistance properties to abrasion and impacts. However, for the blended scoria gravel, the maximum abrasion and impact were achieved at 20/80 scoria to marble mixing ratio, 19.27% < 45%.

#### 3.3.7 Moisture–density relationship of blended scoria gravel and MWA

**Figure 15:**
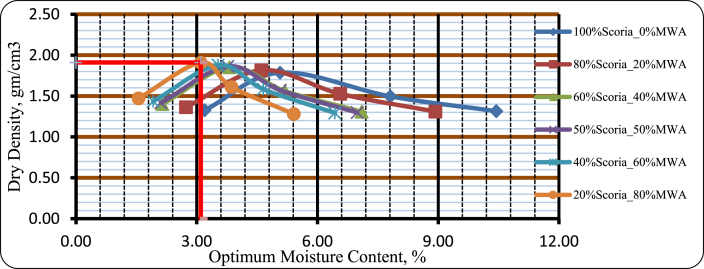
Moisture-density relation curve of blended scoria gravel and MWA.

**Figure 16:**
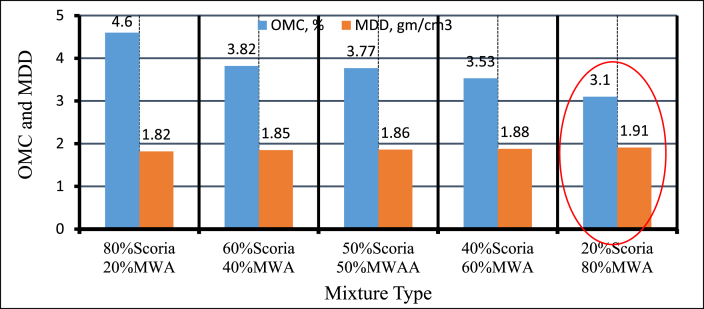
Moisture density relation of blended scoria gravel at different percentages of MWA.

As it was clearly observed from Figure 15, and Figure 16 the moisture content of the mixtures increases with increasing percentages of Scoria gravel, the value increases from 3.10% to 4.60% for Scoria increases by weight from 20% to 80% respectively, that was due to roughness and water holding capacity of scoria. On behalf of maximum dry density test results, the mixture containing high amounts of marble waste aggregate is slightly higher than that of the mixture containing a low amount of MWA. Thus, maximum dry density was increased as a percentage of marble waste aggregate was increased from 1.82 gm/cm^3^ to 1.91 gm/cm^3^ for 80% Scoria–20% MWA and 20% Scoria–80% MWA respectively.

#### 3.3.8 California bearing ratio (CBR) test results of blended scoria gravel and MWA

**Figure 17:**
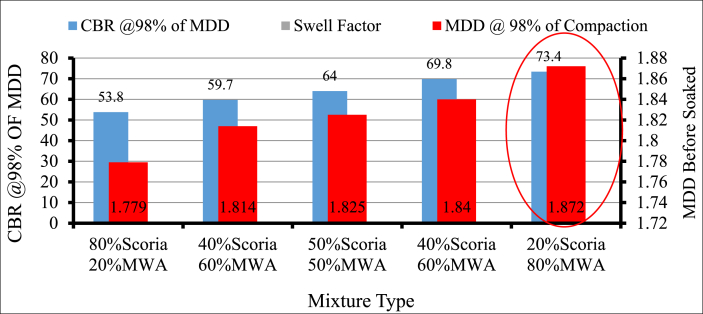
Soaked CBR and MDD test result of blended scoria gravel and MWA

Figure 17 shows the test results for Soaked CBR of Scoria Gravel blended with MWA at different proportions to meet the requirement of ERA standard specification for Mechanically Stable Natural Gravels & Weathered Rocks for use as Base Course Material (GB_2_, GB_3_). The test results of 80% Scoria–20% MWA, 60% Scoria–40% MWA, 50 Scoria–50% MWA, 40% Scoria–80% MWA, and 20% Scoria–80% MWA are 53.80%, 59.70%, 64.0%, 69.80%, and 73.4%, respectively. From the modified scoria gravel by marble waste aggregate the ratio with the best laboratory test results is 20% scoria gravel blending with 80% marble waste aggregate. The obtained engineering properties test results of 20% scoria blended 80% MWA are; 2.56, 1.21%, 17.45%, 13.77%, 21.38%, 132.8 KN, 18.59%, 19.27%, and 73.4%, of SG, FI, EI, ACV, AIV, LAA and CBR respectively.

However, the interesting result was obtained for the engineering property of blended scoria gravel with MWA at a 20/80 percentage by weight ratio as shown in Table 6, but still, the obtained CBR was marginal to use the blended scoria gravel as alternative base course material. This was, due to the high vesicular, rough surface, and porous properties of scoria gravel that make the material weak, and the surface smoothness, flakiness, and Elongated characteristics of MWA that cause the displacement of particles under susceptibility of compaction and movements due to lubrication respectively of soaked CBR test. Therefore, further treatments were conducted by trial and error at a 5% increment of CSA blended with a selected control mixture of 20/80 to improve the CBR value of blended scoria gravel.

**Table 6:** Summary of results conducted on scoria gravel and marble waste aggregate samples.

Aggregate Test Type	Percentage of Scoria gravel blended with MWA mixing proportion at 20% MWA increments					ERA Standard Specification
	20% MWA	40% MWA	50% MWA	60% MWA	80% MWA	
OMC%	4.6	3.82	3.77	3.53	3.1	NS
MDD (g/cc)	1.81	1.85	1.83	1.88	1.91	NS
CBR (%)	45.8	59.7	64	69.8	73.4	>80%
Water Absorption (%)	4.43	3.36	2.83	2.29	1.21	<2
Specific Gravity	2.3	2.38	2.43	2.47	2.56	>2.5
ACV (KN)	29.5	26.1	24.7	23.4	21.4	<29%
TFV (KN)	71.5	75.7	81.6	100	133	>110 KN
AIV	24.44	22.83	20.93	19.56	18.59	<25%
Atterbegr limit	NP	NP	NP	NP	NP	<6
Loss Angeles Abrasion	24.8	22.9	22	21.1	19.3	<45%
FI	14.44	15.38	15.87	16.38	17.45	<30
EI	12.17	12.67	12.93	13.2	13.77	10–35%

### 3.4 The properties of scoria gravel, MMA, and CSA

#### 3.4.1 Particle size distribution

**Figure 18:**
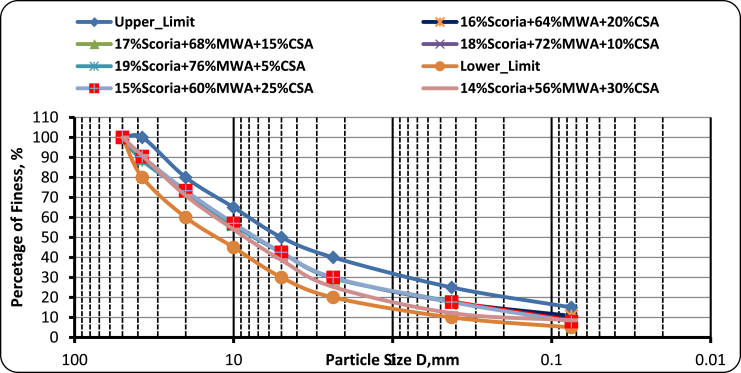
Test result for particle size distribution curve of scoria gravel, MWA, and CSA.

Table 7 and Figure 18 was shown that the particle size distribution was normal with ERA standard specification upper and lower boundary envelope lines at all mixing ratios. From the test conducted on scoria gravel blended with MWA and CSA, the increasing amount of CSA by a trial and error percentage by weight at 5%, 10%, 15%, 20%, 25%, and 30% CSA improves the gradation and the optimum proportioning was determined to fulfill the ERA standard specification manual requirement.

**Table 7:** Particle size distribution test result for scoria gravel, MWA, and CSA.

Sieve Size in, mm	Percentage of Passing IS Sieve at Different Percentage of Mixture						ERA Standard Specification for GB2 and GB3	
	19% Scoria + 76% MWA + 5% CSA	18% Scoria + 72% MWA + 10% CSA	17% Scoria + 68% MWA + 15% CSA	16% Scoria + 64% MWA + 20% CSA	15% Scoria + 60% MWA + 25% CSA	14% Scoria + 56% MWA + 30% CSA		
50	100.00	100.00	100.00	100.00	100.00	100.00	100	100
37.5	89.22	89.68	90.14	90.60	89.60	90.79	80	100
20	71.12	71.34	71.55	71.77	69.57	70.89	60	80
10	54.46	54.75	55.05	55.35	53.21	53.96	45	65
5	40.63	40.75	40.88	41.02	37.27	38.62	30	50
2.36	27.67	27.84	28.02	28.22	24.49	25.24	20	40
0.425	16.21	16.20	16.19	16.21	12.29	12.18	10	25
0.075	6.81	6.71	6.62	9.20	8.01	8.59	5	15
Pan	0.00	0.00	0.00	0.00	0.00	0.00

#### 3.4.2 Moisture–density relationship of blended scoria gravel, MWA and CSA

**Figure 19:**
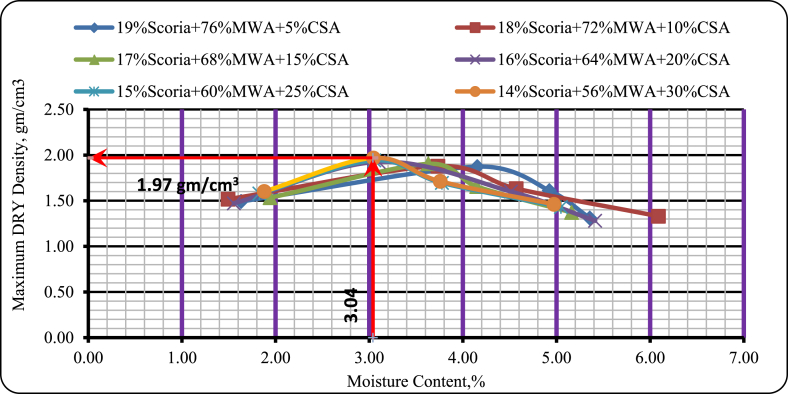
Moisture density test results of scoria, MWA, and CSA

As it was seen in Figure 19, the optimum moisture content of the mixtures increases with increasing percentages of convention aggregate. The maximum dry density obtained from test results was 1.97 gm/cm^3^ attained at 3.04% of optimum moisture content. Besides this, the amount of water decreases from 4.15% to 3.04%. Thus, the amount of water depends on the increasing or decreasing amount of Scoria gravel value in the mixture. Simultaneously, the dry density was increased from 1.87 gm/cm^3^ to 1.97%. From the result, we can say that the value for dry density was increased with increasing conventional aggregate.

#### 3.4.3 California Bearing Ratio (CBR) test results of scoria gravel, MWA, and CSA

**Figure 20:**
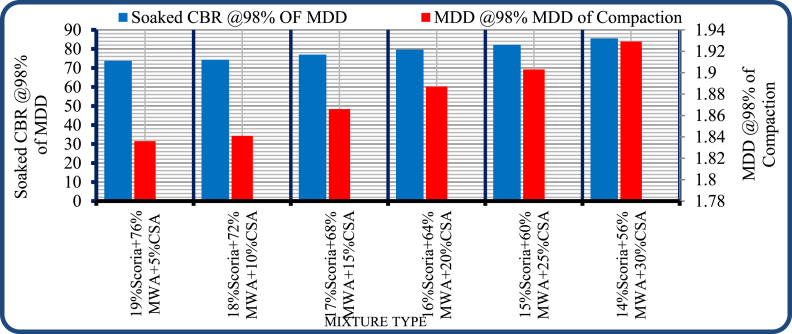
Soaked CBR test result of scoria, MWA, and CSA

Based on the test result shows on Figure 20 the CBR value were increased thus, 73.70%, 74.30%, 77.00%, 79.60%, 82.13% and 85.50% are at 19:76:5, 18:72:10, 17:68:15, 16:64:20, 15:60:25 and 14:56:30 blending ratio of scoria gravel to marble waste aggregate to crushed stone aggregate percentage by weight respectively. Hence, the CBR value of scoria gravel was satisfy the minimum soaked CBR value of ERA standard specification at 15 Scoria–60% MWA–25%CSA and 14% Scoria–56% MWA–30% CSA blending points of percentage by weight. It was concluded that up to 15% scoria gravel_60% MWA_25% CSA uses as an alternative base course material.

## 4 Conclusions

Based on the results acquired in the laboratory, the following conclusions have been drawn:

- Particle size distribution of Scoria Gravel and Marble Waste Aggregate does not fulfill the ERA standard specification for (GB_2_ and GB_3_). According to AASHTO soil classification system, all materials were classified as granular material A-1-a, and as per USCS, the materials were classified as well-graded gravel with sand and silt or clay GW-GM after crushing.
- The physical and mechanical engineering property of aggregate, including particle size distribution test results of neat scoria gravel, was proven to the marginality of material to utilize as an alternative base course construction material without modification. Based on specific gravity test results of scoria gravel, MWA, and CSA, the SG of scoria was lower than that of MWA and CSA, as scoria gravel was light in weight when compared to both materials. Even though, the SG of MWA is less than CSA it satisfies the ERA minimum requirements.
- The Water Absorption of Scoria gravel was much higher than MWA and CSA, this is due to scoria gravel being high in Porous, Rough Surface, and Vesicular materials. The WA of MWA was much lesser than CSA; this was also due to the Smoothness and Very Low Porosity of MWA. The blended scoria gravel at 20% increments of MWA samples satisfies all the required quality for GB_2_ and GB_3_ base course materials at 20/80 percentage by weight of scoria to MWA ratio. However, the CBR does not satisfy the ERA specified value of 73.4% < 80% for base coarse pavement construction materials.

Hence, the blending of control mixture with CSA has done by trial and error, at 5%, 10%, 15%, 20%, 25% and 30% percentage by weight of CSA was obtain the CBR of 73.7%, 74.3%, 77%, 79.6%, 82.13% and 85.5% respectively. The satisfactory CBR values were obtained at 25% CSA.

Finally, the use of scoria gravel, MWA, and CSA up to 15:60:25 percentage by weight proportion respectively, when it was compared with ERA requirements and when it was found near to construction site and in places where scoria gravel was abundantly available might help to meet reduces the extraction of conventional aggregates, and slow down any detrimental effects on the environment. Further research should be conducted to evaluate the long-term effects and performance of scoria gravel at the base course layer on the durability and resilient modulus of pavement structures with full-scale road tests.

## Declarations

### Author contribution statement

Multazem Mohammed: Conceived and designed the experiments; Performed the experiments; Analyzed and interpreted the data; Wrote the paper.

Murad Mohammed, Abubekir Jemal & Anteneh Geremew: Conceived and designed the experiments; Analyzed and interpreted the data; Contributed reagents, materials, analysis tools, or data; Wrote the paper.

### Funding statement

This work was supported by Jimma Institute of Technology.

### Data availability statement

The data that has been used is confidential.

### Declaration of interest's statement

The authors declare no conflict of interest.

### Additional information

No additional information is available for this paper.
